# A New Aural Douche

**Published:** 1880-11-20

**Authors:** C. R. Upson

**Affiliations:** Surgeon to the Department of Nose, Throat and Lung Diseases, Atlanta Hospital, Atlanta, Ga.


### A NEW AURAL DOUCHE.

BY C. R. UPSON, M.D,

Surgeon to the Department of Nose, Throat and Lung Diseases, Atlanta Hospital, Atlanta, Ga.

Having frequent occasion, in the treatment of aural and nasal affections, to apply plain and medicated solutions to these parts, and appreciating the comfort of the patient, to say nothing of the benefits of maintaining the solution used at a uniform temperature during the whole period of its application, I have devised an apparatus which fulfills this indication.

It consists essentially of a glass reservoir of three pints' capacity, that is placed in a copper water-bath, provided with a thermometer in its top, and this in turn is enclosed in a sheet-iron jacket containing a spirit-lamp, the tube of which is provided with an outer cylinder by which to regulate the size of the flame. The solution is forced from the reservoir by means of an atomizing bulb and tubing attached to a glass tube passing through a rubber stopper to the bottom of the vessel. The apparatus is provided with nozzles for applying the solution to the ear and posterior nares..

I would briefly call the attention of the profession to the advantages claimed for this apparatus :

1. The solution being contained in a *glass* reservoir may be medicated with any remedial agent which the physician may elect.

2. The solution may be maintained at any desired temperature, indicated by the thermometer in the top of the bath, by means of the spirit-lamp.

3. The capacity of the reservoir, three pints, being sufficient for any application which may be required at one sitting.

4. It may be used, by attaching a longer piece of tubing, and placing the apparatus in an elevated position, as a syphon.

5. Its simplicity in construction, there being no complicated parts to become disarranged.

In addition to the uses already named, as an aural and nasal douche, it will be found a valuable means of applying solutions to the mucous membrane of the bladder, vagina, and even to the cavity of the uterus itself. I have for a considerable time been using this apparatus, though in a rude form, and have found it so useful that I have had a more perfect one made for me by Messrs. Codman & Shurtleff, instrument makers, Boston, who will supply such other members of the profession as may desire one.

Since penning the above, a troublesome patient has impressed upon my mind the advisability of having the free use of both hands while syringing the ear, in order to secure which, I have bored a second hole in the rubber stopper of the reservoir, through which I have inserted a tube (of the same size and shape as that which accompanies the douche) but a short distance, not allowing it to touch the solution. To the upper end of this tube I have attached an ordinary atomizing bulb, with a sufficient length of tubing to allow of its resting upon the floor, thus enabling me to compress it with my foot.

